# Development of Playfulness in Children with Low Executive Functions: The Role of Parental Playfulness and Parental Playtime with Their Child

**DOI:** 10.3390/bs14070542

**Published:** 2024-06-27

**Authors:** Isabelle Duss, Cornelia Ruedisueli, Corina Wustmann Seiler, Patricia Lannen

**Affiliations:** 1 Marie Meierhofer Children’s Institute (MMI), Associated Institute of the University of Zurich, 8005 Zurich, Switzerland; lannen@mmi.ch; 2 Department of Pre-Primary and Lower Primary Level and Department of Research & Development, Zurich University of Teacher Education (PH Zurich), 8090 Zurich, Switzerland; cornelia.ruedisueli@phzh.ch (C.R.); corina.wustmann@phzh.ch (C.W.S.)

**Keywords:** children’s playfulness, low executive functions, parental playfulness, parental playtime, longitudinal study, repeated-measures hierarchical linear models

## Abstract

Children with low executive functions (EFs) are described as having lower levels of playfulness, the quality of children’s play, compared to children with EFs within the normal range. However, how playfulness in children with low EFs develops over time remains unclear. Additionally, little is known about how parental playfulness and parental playtime with their child affect these developmental trajectories in children with low EFs. To address these research gaps, we measured playfulness in 62 children with low EFs and 62 children with EFs within the normal range aged 3 to 6 years at three time points over 2 years. We used the Children’s Playfulness Scale, which captures multi-informant perspectives from parents and teachers. Moreover, the parents of children with low EFs reported their own playfulness and their playtime with their children at T1. Repeated-measures hierarchical linear models indicated significantly lower levels of playfulness in the children with low EFs than in the controls, with no significant changes observed over 2 years in either group. In the children with low EFs, we found a significant positive relationship between parental playfulness at T1 and children’s playfulness 2 years later but a significant negative relationship between parental playtime at T1 and children’s playfulness 2 years later. These results prompt a broad discussion on potential implications for the enhancement of playfulness in children with low EFs within the family environment.

## 1. Introduction

Children’s playfulness, defined as joy, motivation, and engagement in play [1], is of great importance for children’s well-being [2] and helps children to approach new situations. Children’s executive functions (EFs) are central to targeting their thoughts and actions during play. EFs enable actions to be planned in advance, obstacles to be overcome, and long-term goals to be pursued [3]. Various studies have described children with developmental delays that affect their EFs as having lower levels of playfulness than children without developmental delays [4,5,6]. Nevertheless, the developmental trajectories of playfulness in children with low EFs have not yet been compared to those of children with EFs within the normal range. Given the great importance of parenting to young children’s development [7,8], understanding the role of parents in shaping the developmental trajectories of playfulness is essential. Thus, it is crucial for parents to cultivate an environment conducive to their children’s overall development and in particular to their playfulness. This aspect of parental influence is especially important for children with low EFs, as these children are especially dependent on parental support [9]. Initial studies have also indicated that parental supportiveness, for example, is positively related to children’s playfulness [10]. However, it is crucial to investigate whether parental playfulness and parental playtime with their child influence the developmental trajectories of playfulness in children with low EFs. 

### 1.1. Playfulness

Children’s playfulness is described as the quality of children’s play [1,11] and can be understood as a resource for lifelong learning [12]. Initial research into children’s playfulness was conducted by Lieberman [13], who defined the five dimensions of children’s playfulness: (1) physical spontaneity is the level of movement and coordination during play; (2) cognitive spontaneity describes the imaginary aspects of the children’s play and their willingness to slip into different roles while playing; (3) social spontaneity is the children’s flexibility in dealing with other children and their willingness to initiate play activity and to cooperate with others; (4) manifest joy is the children’s ability to express joy and enthusiasm during play; and (5) sense of humor refers to the children’s ability to fool around, gently tease others, and joke with other children. These five dimensions describe the holistic nature of children’s play behavior. Barnett [1] continued Lieberman’s work and developed the Children’s Playfulness Scale (CPS), a reliable and valid instrument for assessing children’s playfulness from ratings by teachers and parents. Various studies have demonstrated the relationship between playfulness and children’s creativity, coping, adaptability, and social skills [10,14,15,16,17]. Furthermore, a positive cross-sectional relationship was found between children’s playfulness and their EFs [18].

### 1.2. Executive Functions and Playfulness

EFs are higher, self-regulatory, cognitive processes that control thoughts and actions [3]. EFs can be categorized into three domains: inhibition control, working memory, and cognitive flexibility [19]. These three domains exhibit different developmental trajectories [20]. Whereas inhibition control develops primarily in children under 5 years of age [20], significant development in working memory and cognitive flexibility often begins around the age of five and continues into adolescence [21]. EFs are of central importance to cognitive and social development, and they are also reliable predictors of later school success [22,23]. The studies of children’s play have shown that preschool children with well-developed EFs show more fantasy play and more extensive pretend play than children with lower EFs [24,25]. Moreover, EFs are related to social competencies [26] and therefore play a crucial role in social play interactions. Taking turns, negotiating roles, and following the rules of a game all require well-developed EFs. 

Neurodevelopmental disorders such as attention deficit hyperactivity disorder (ADHD) and autism spectrum disorders (ASDs) are associated with reduced EFs [27,28,29]. Various cross-sectional studies have shown that children affected by such disorders exhibit lower levels of playfulness than typically developing children [4,5,6,30]. These studies involved comparisons between 20 to 30 children with neurodevelopmental disorders and 20 to 30 typically developing children at different ages from 3 months to 14 years. The level of playfulness in these studies was assessed during live observations. The authors offer various explanations for the differences in the levels of playfulness between the groups. For example, Leipold and Bundy [4] reported that children with ADHD have difficulties in joining a group, initiating play with other children, and playing cooperatively, and these difficulties are reflected in the lower levels of playfulness. The lower levels of playfulness in children with ASDs may be associated with their challenges with communication, social engagement, and imaginative play [6,30]. 

### 1.3. Development of Children’s Playfulness

To date, little is known about whether playfulness is stable or changes over time during early childhood. Furthermore, the potential predictors that affect development trajectories in children’s playfulness remain unexplored. In the initial investigations of children’s playfulness, Lieberman [13] and Barnett [1] assumed that children’s playfulness remains stable over time and settings. In contrast, current research on children’s playfulness tends to assume that children’s playfulness can be enhanced or limited by external factors including home environment and institutional settings [11]. Studies have demonstrated setting effects: for example, children’s playfulness varies depending on whether children are interacting with their teacher or their parents [5]. Other studies have shown positive intervention effects in increasing playfulness in young children [30,31]. 

Cross-sectional studies provide indications of a possible increase in children’s playfulness levels in early childhood. These studies showed positive correlations between playfulness and age in children between 2 and 8 years [14,32]: the older the children become, the more playful they are. To date, however, there are hardly any representative longitudinal studies on the development of playfulness in childhood, and the few available are often hindered by very small sample sizes [33,34]. Waldman-Levi et al. [35] provided initial indications that playfulness increases in very young children between the ages of 6 and 24 months.

### 1.4. Parental Playfulness

In his seminal socio-ecological model, Bronfenbrenner [36] emphasized the pivotal role parents play in children’s development. Consequently, the home environment exerts a substantial influence on children’s development and play behaviors [7,8]. The relationship between children’s playfulness and the aspects of the quality of parent–child interactions, such as responsiveness, sensitivity [37,38], and parental playfulness [32,39], indicate the central role of parents in the development of children’s playfulness. Parents act as role models when playing with their children. If parents are playful themselves and spend time with their children, the children are encouraged to be playful too [40]. Playfulness in adults is reflected more in daily life than in play activities, yet it is associated with well-being, physical fitness, and higher life satisfaction [41]. Parental playfulness refers to parents’ ability to engage with their children playfully. For example, in parent–child interactions, parental playfulness manifests itself in parents’ ability to reframe different situations with their children in a playful way, to turn difficult situations into fun, and to remain flexible and humorous during stressful times [42,43]. This is particularly important for children with low EFs, who may find certain tasks more challenging. Here, parental playfulness can provide a relaxed play environment in which these children can develop their social and cognitive skills. Furthermore, parental playfulness has been shown to be positively associated with children’s playfulness [32,39]. Moreover, the parents with higher levels of playfulness reported fewer emotional regulation difficulties in their children [43] and less child negativity [44]. 

### 1.5. Parental Playtime with Their Child

In addition to parental playfulness, parental playtime may also affect children’s playfulness. The parents who are actively engaged in children’s play can foster a rich play environment for their children [45]. Play interactions with parents have been identified as especially beneficial for the overall development of children with developmental delays [46]. In these play interactions, the parents can use their understanding of their children’s needs and challenges and integrate this knowledge into play situations. Parents are able to structure play interactions by facilitating social skills, social communication, and learning opportunities for their children [47]. To the best of our knowledge, hardly any studies have specifically investigated the relationship between child development and the time parents spend playing with their children. Gardner [48] was able to demonstrate that increased parental playtime with their child correlates with reduced behavioral problems in such children. However, how parental playtime with their child affects their playfulness has not yet been investigated either in children with low EFs or in children with EFs within the normal range.

### 1.6. Summary and Research Questions

In conclusion, children with low EFs are described as having lower levels of playfulness than their typically developing peers. However, the development trajectories of playfulness in children with low EFs in comparison to children with EFs within the normal range remain unstudied. Furthermore, the impact of parental playfulness and parental playtime on the developmental trajectories of playfulness in children with low EFs remains unclear. Because children with low EFs benefit particularly from parental support [9], investigating the effects of parental playfulness and parental playtime on these children seems especially important. 

Therefore, this study pursued two objectives. Firstly, we investigated how playfulness changes over a 2-year period in children with low EFs compared to children with EFs within the normal range. Secondly, we examined how parental playfulness and parental playtime were related to the playfulness of children with low EFs both cross-sectionally at T1 and longitudinally across three time points. 

From the body of research discussed above, we assumed that children with low EFs exhibit lower levels of playfulness than do their counterparts with EFs within the normal range at T1 (H1). Additionally, we hypothesized that the playfulness of both groups of children increases over the two years (H2a), but significantly less in children with low EFs than in those with EFs within the normal range (H2b). Given the positive relationship between parental playfulness and children’s playfulness [32,39], coupled with evidence that parental playtime counteracts children’s behavioral problems [48], we assumed that both parental playfulness and parental playtime with children exert a positive effect on playfulness in children with low EFs (H3a) cross-sectionally at T1. Furthermore, we expected that parental playfulness and parental playtime contribute positively to the development of playfulness in children with low EFs (H3b).

## 2. Materials and Methods

### 2.1. Procedure

The present study was part of the “Playfulness in early childhood: A longitudinal study of individual and contextual determinants (Playful)” project. The Ethics Committee of the Faculty of Philosophy at the University of Zurich, Switzerland, reviewed and approved the study (ethics approval number 20.12.13). 

In this project, 81 teachers from 34 childcare centers and 47 kindergartens in 12 cantons in the German-speaking part of Switzerland provided their written consent to participate in the project. With their consent, the teachers agreed to forward information about the study to the parents and, when they had received parental consent, complete a questionnaire about each participating child in their classroom. Note that in Switzerland, childcare centers are privately funded and privately organized and are offered for children from infancy until they enter kindergarten. In contrast, kindergartens are part of the public school system in Switzerland for children between the ages of four and six.

In total, 848 parents agreed to complete a questionnaire and that the research team could conduct a short test of EFs with their child in the childcare center or kindergarten. To participate in the testing, the children had to be at least 3 years old, to be present on-site at the childcare center or kindergarten on the day of the testing, and to provide their verbal assent to being included in the study. Within these criteria, the testing of executive functions was conducted with 498 children. EFs were measured in spring 2021 at T1. The parents and teachers provided information on the children’s playfulness in spring 2021 (T1), spring 2022 (T2), and spring 2023 (T3). Each parent provided additional information about their child, family demographics, their playfulness, and their playtime with their child at T1. The parents’ and teachers’ questionnaires were administered online with the Survalyzer software. 

The teachers, parents, and children were informed that their participation was voluntary, that all the data would be anonymized after the survey, that these data would only be used for scientific purposes, and that withdrawal from the study was possible at any time without providing any reason. 

### 2.2. Participants

The EFs of 498 children aged between 3 and 6 years were measured with the Minnesota Executive Function Scale App [49] at T1. Of these, 62 children (12.4%) achieved a test score of −1 standard deviation or lower. These children formed the group of children with low EFs. Of the children who scored within a standard deviation of between −1 and +1, we randomly selected 62 children to form the group of children with EFs within the normal range. A total of 124 children were on average 5.42 years old (SD = 1.08), and 37% were female: 27% in the group of children with low EFs and 47% in the group of children with EFs within the normal range. The demographic characteristics of the two groups are shown in Table 1. Some 37 children from the group of children with low EFs and 41 children from the group with EFs within the normal range had Swiss nationality. Among the mothers in the group of children with low EFs, 10 had at least an academic degree, as did 26 mothers in the group of children with EFs within the normal range. The two groups did not differ significantly in children’s age (*t*(122) = −0.315, *p* = 0.753), but they differed significantly in the distribution of gender (*t*(122) = −2.259, *p* < 0.05) and as expected, they differed significantly in their EF z-scores (*t*(95.591) = −15.580, *p* < 0.001).

### 2.3. Measurement Instruments

*Children’s Playfulness:* Children’s playfulness was assessed with the Children’s Playfulness Scale (CPS; [1]) in the German version [32]. The CPS contains 23 items using a 5-point Likert scale (1 = does not sound at all like the child; 5 = sounds exactly like the child). The 23 items are distributed across the five dimensions defined by Lieberman [13]: (1) physical spontaneity (four items, e.g., “The child’s movements are generally well-coordinated during play activities”), (2) cognitive spontaneity (four items, e.g., “The child invents his/her own games to play”), (3) social spontaneity (five items, e.g., “The child plays cooperatively with other children”), (4) manifest joy (five items, e.g., “The child expresses enjoyment during play”), and (5) sense of humor (five items, e.g., “The child enjoys joking with other children”). Both the parents and teachers completed the questionnaire on children’s playfulness at all three measurement time points. For a multi-informant approach, their reports were combined to calculate one mean for each item. The mean values were then estimated for each dimension, and the resultant manifest total playfulness score was derived as the mean of these five dimensions. The internal consistency for the total playfulness scores was very good, with Cronbach’s Alpha α_T1/T2/T3_ = 0.86/0.87/0.87, and the test–retest reliability was *r*_T1,T2/T2,T3_ = 0.65/0.55 (*p* < 0.001) for our sample. In the present study, we assessed children’s playfulness using the parent and teacher reports for all three measurements, as children’s play behavior can vary depending on the environments and play partners. By averaging the ratings of the parents and teachers, the global playfulness of each individual child can be captured more validly, and the parent and teacher biases can be minimized [50].

*Executive Functions:* Executive functions were measured using the Minnesota Executive Function Scale App ([MEFS AppTM; [49]), an adaptive virtual sorting task administered to children from the age of 24 months via a tablet. The processing time for this task varied between 2 and 6 min. The MEFS app was tested with a sample of 35,000 American children and can be seen as a reliable [51] and valid [52] assessment tool. Validity was also confirmed for clinical samples [53,54,55]. The MEFS yields a total score for each child, incorporating both the score achieved and the corresponding reaction time. The children were tested by research assistants, who all were trained and certified in the standardized use of the MEFS by Reflection Science, the developers of the MEFS. A German translation of the application was available. To distinguish the children with low EFs from the children with EFs within the normal range, the total score was z-standardized separately for each age group: 3 years old, 4 years old, 5 years old, and 6 years old. 

Parental Playfulness: Parental playfulness was assessed with the Short Measure for Adults’ Playfulness (SMAP; [56]) at T1. The SMAP contains five items (e.g., “I often do playful things in my everyday life”) and is assessed on a 7-point Likert scale (1 = not at all true; 7 = totally true). An overall mean score was calculated from the five items. The scale showed very good internal consistency for the present study (α = 0.88). 

*Parental Playtime with their Child:* The parents indicated on a 5-point Likert scale (1 = less than 0.5 h; 5 = more than 4 h) how much time they play with their child on average per weekend day at T1. We asked about the weekend because we presume that the parents’ available time to engage in play activities with their children is more consistent across families during weekends compared to weekdays. This suggests that the actual time the parents spend playing with their children is more comparable on weekends than during weekdays. 

*Demographic Characteristics:* The parents provided information at T1 on demographic characteristics of the child and the family: the child’s age and gender, family nationality and language, and the parents’ education degree. 

### 2.4. Data Analysis

Statistical analyses were conducted in R [57] using the lme4 package [58] to perform repeated-measures hierarchical linear models (RM-HLM) with contrast effects. First, descriptive statistics including intercorrelations were calculated separately for the children with low EFs and the children with EFs within the normal range for all the study variables. To analyze developmental trajectories in playfulness between T1 and T2 and between T1 and T3, repeated contrasts were modeled and included in the RM-HLM [59]. Parental playfulness and parental playtime with their child were centered for easier interpretation of the results. In all the models, the children’s genders and ages, also centered, were included as control variables due to their predictive significance in previous studies [32,60,61]. 

## 3. Results

### 3.1. Descriptive Statistics 

The means and standard deviations for all the outcome variables are presented in Table 2 separately for the children with low EFs and the children with EFs within the normal range. Both groups of children were rated lowest in the social spontaneity dimension and highest in the manifest joy dimension at all three time points. 

Intercorrelations for the total playfulness scores at all three time points, age, and gender were calculated separately for the children with low EFs and the children with EFs within the normal range and are provided in Table 3. In addition, intercorrelations with parental playfulness and parental playtime with their child were also calculated for children with low EFs. The intercorrelations showed significant, positive, and large relationships between total playfulness scores at all three time points for the children with low EFs. For the children with EFs within the normal range, a significant, moderate to large, and positive correlation occurred between the children’s total playfulness scores at all three time points. In the children with low EFs, parental playtime at T1 exhibited a significant, moderate, and negative correlation with the children’s total playfulness scores at T1 and T2. There was a low but insignificant positive correlation between parental playfulness and the children’s total playfulness scores at all three time points. No correlation was found between gender and children’s total playfulness scores at any of the three time points for either the children with low EFs or those with EFs within the normal range. A significant, moderate, and positive correlation was found between age and the total playfulness score in the children with EFs within the normal range at T1.

### 3.2. Cross-Sectional Differences in Playfulness between the Two Groups

Table 4 shows a significant difference between the two groups at T1 in both the total playfulness score and the dimensions of social spontaneity, cognitive spontaneity, and sense of humor. In contrast, no significant differences were found in the dimensions of manifest joy or physical spontaneity between the two groups at T1. 

### 3.3. Developmental Trajectories in Children’s Playfulness

There was no significant change in the total playfulness scores from T1 to T2 or from T1 to T3 for children with low EFs or for children with EFs within the normal range. However, Table 4 shows a significant increase in the social spontaneity dimension from T1 to T3 and a significant increase in the sense of humor dimension from T1 to T2. No change was observed for the remaining three dimensions of children’s playfulness over the 2-year period. 

When examining whether a possible change over time depends on the group (see Table 5), no significant interaction effects were observed for either the total playfulness score or any of the five dimensions. This indicates that there was no recognizable change in the overall playfulness or in physical spontaneity, cognitive spontaneity, or manifest joy for either group. Moreover, both social spontaneity and sense of humor exhibited similar increases in both groups.

### 3.4. Effects of Parental Playfulness and Parental Playtime on Playfulness in Children with Low EF

Table 6 shows that playfulness in the children with low EFs was significantly positively related to parental playfulness and significantly negatively related to parental playtime. This implies that at the initial baseline (T1), playful parents tend to have more playful children than parents who rated themselves as less playful. But, the children with low EFs tend to receive lower ratings in playfulness as parental playtime with them increases. 

### 3.5. Effects of Parental Playfulness and Parental Playtime on the Developmental Trajectories in Playfulness in Children with Low EFs

**Figure 1:**
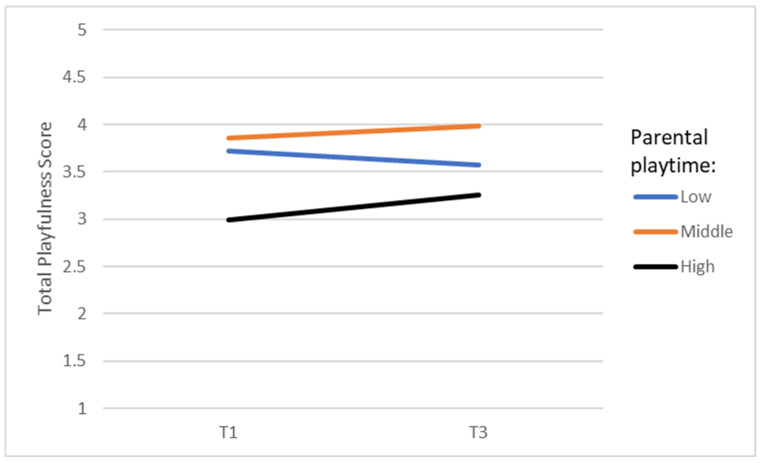
Time × parental playtime interaction.

A two-way interaction was calculated to examine whether changes might have occurred in children’s playfulness over the 2-year period depending on parental playfulness and parental playtime with their child. No significant interaction was found between time and parental playfulness (see Table 7). However, a significant interaction was found between time and parental playtime with their child (see Table 7 and Figure 1). This means that playfulness increased significantly over the 2-year period in the children with low EFs when these parents played more often with their children. 

## 4. Discussion

This study is the first to compare the developmental trajectories of playfulness in young children with low EFs with controls in a longitudinal study with three measurement time points distributed over 2 years involving over 120 participants. Additionally, the study examined cross-sectional and longitudinal relationships between children’s playfulness and parental playfulness and parental playtime with their children with low EFs.

### 4.1. Cross-Sectionally Differences in Playfulness

The first hypothesis that children with low EFs exhibit lower levels of playfulness at baseline than children with EFs within the normal range, can be confirmed. In addition, the children with low EFs demonstrated significantly lower scores in three of the five dimensions of children’s playfulness: cognitive spontaneity, social spontaneity, and sense of humor. This suggests that EFs are primarily related to those dimensions of playfulness that include cognitive and social competencies. These results align with those of other studies showing that EFs are linked to children’s cognitive development [54], school success [22,23], and social skills [26]. 

Nevertheless, the two groups of children did not differ in two dimensions of playfulness, physical spontaneity and manifest joy. The fact that physical spontaneity does not differ between the two groups contrasts with the findings of studies that have looked at the relationship between children’s EFs and motor skills, which assume a positive correlation [62,63]. However, the physical spontaneity dimension of the CPS [1] primarily assesses the quantity of physical movements during play rather than their quality. This distinction suggests that the physical spontaneity dimension measures something other than motor skills, which may explain the lack of differences between the two groups in quantity. Additionally, we found no differences between the two groups in the manifest joy dimension of the CPS [1]. Whereas studies report an association between EFs and children’s emotion regulation [64,65], the manifest joy dimension assesses the expression of such emotions as happiness and enthusiasm rather than emotional regulation. 

### 4.2. Developmental Trajectories in Playfulness

Hypothesis 2a, that the playfulness of both groups of children increases over two years, could not be confirmed in the present study. The total playfulness scores appeared to be stable for the age group surveyed, both in children with low EFs and in children with EFs within the normal range. These findings concur with Lieberman’s [13] and Barnett’s [1] original thoughts. However, a current study with about 800 children without developmental delays shows that development trajectories of playfulness differ depending on the age of the children and on the individual dimensions of children’s playfulness [66]. Unfortunately, the sample size of children with low EFs in this study prevents detailed analysis. Consequently, a larger sample size would prove advantageous in facilitating differentiated analyses of distinct age groups. An age classification might enable specific patterns and trends to be identified in relation to developmental trajectories in playfulness and to verify the results of the study by Wustmann Seiler et al. [66] are also valid for children with low EFs.

When we examine the individual dimensions of children’s playfulness, we do not see any changes in either group in cognitive spontaneity, physical spontaneity, or manifest joy. However, we found increases in social spontaneity and sense of humor in both groups. This increase could be an expression of children’s general socio-emotional development. Children’s social play activities evolve with the onset of the theory of mind, a development that primarily occurs between the ages of 3 and 5 years [67]. During this period, play becomes progressively more social and is directed towards the needs of other children [68]. In addition, peers become increasingly important to children from the age of 3 years [69], when social play increasingly replaces solitary and parallel play [70].

### 4.3. Effects of Parental Playfulness and Parental Playtime on Playfulness in Children with Low EFs Cross-Sectionally

In Hypothesis 3a, we expected that parental playfulness and parental playtime with their child exert a positive effect on playfulness in children with low EFs at T1. This hypothesis was only partially confirmed. We found a significant and positive relationship between children’s playfulness and parental playfulness but a significant and negative association between children’s playfulness and parental playtime with their child.

In this study, parental playfulness positively predicts children’s playfulness in children with low EFs. Already Bandura [71] observed that children learn a lot through observations and imitations. Parents who are playful and creative may encourage their children to imitate such behavior. This not only leads to greater playfulness in children with low EFs but likely also creates a positive atmosphere in the family [39]. A positive environment can provide children with the confidence to express themselves through play. 

Contrary to our initial hypothesis 3a, we found a negative relationship between parents’ playtime with their children and playfulness in children with low EFs: the less playful the child was, the more parents played with them. We assume that parents recognize that children with low EFs, who are less playful, profit from more parental support during their play. These results concur with other studies showing that parents of children with developmental impairments see their children’s needs and respond appropriately to them [72,73]. 

### 4.4. Effects of Parental Playfulness and Parental Playtime on the Development Trajectories in Playfulness in Children with Low EFs

In Hypothesis 3b, we expected that parental playfulness and parental playtime with their child contribute positively to the developmental trajectories in playfulness of children with low EFs. This hypothesis was again only partially verified. Parental playfulness had no notable effect over time on developmental trajectories of playfulness in children with low EFs. In contrast, the amount of time parents played with their children affected the developmental trajectories of children’s playfulness over the 2-year period: the more parents played with their children, the more these children’s playfulness increased. It is noteworthy that the children who initially demonstrated the lowest level of overall playfulness seemed to derive the greatest benefits from parental involvement in their play. It is possible that children who were not very playful in the beginning had the greatest potential for improvement so that their parents’ effort paid off. 

### 4.5. Strengths and Limitations

This study was the first to longitudinally compare the developmental trajectories of playfulness in children aged 3 to 6 years with low EFs with those of children with EFs in the normal range. Furthermore, the study investigated the effects of parental playfulness and parental playtime on the development trajectories of children’s playfulness in children with low EFs. Standardized measures were used from different perspectives, including parent and teacher reports and direct testing of EFs with children in their childcare center or kindergarten. 

The current sample reflects the educational background of mothers in Switzerland quite well [74], and executive functions are known to be related to parental educational background [75,76]. Moreover, the proportion of Swiss children in our study is representative of the total population of 3 to 8-year-old children in Switzerland [77]. 

In addition to these strengths, certain limitations require acknowledgment. Firstly, because the original standardization of executive functions with the Minnesota Executive Function Scale App [49] was performed in the United States of America, we had to conduct a new standardization specifically for the present study. Future research might benefit from a standardization with a Swiss sample. Secondly, the evaluation of children’s playfulness was reliant on parents’ and teachers’ observations. Enhancing the robustness of the findings might involve complementing this subjective assessment with objective measures, such as external observations. Expanding the methodology to include a range of assessment methods would contribute to a more nuanced understanding of children’s playfulness and its development. Thirdly, a larger sample would strengthen the generalizability of future studies’ findings and allow for a more extensive exploration of patterns in developmental trajectories of children’s playfulness. It would also enable more complex analyses, such as the latent modeling of playfulness and separate age group analyses. Fourthly, the current results may not be applicable to children who do not attend institutional settings. Children attending childcare centers or kindergarten may have more opportunities to play with other children, navigate conflicts with peers, and learn about social norms and rules, which are crucial for their social-emotional development [78]. In Switzerland, compulsory schooling begins at the age of 4 or 5 years, and only about 35% of all pre-school children attend a childcare center [79]. Consequently, the inclusion of educational settings and the home environment in subsequent research would provide a better understanding of the breadth of applicability of our findings. Fifthly, only the quantity of parental playtime was recorded in this study and not the quality. A more in-depth analysis of how parents play with their children and an understanding of the quality of interaction during play could provide further insights into the relationship between parental playtime and children’s playfulness. Sixthly, parental playfulness was measured using the SMAP [56]. This scale measures the tendency of parents to be playful and does not specifically refer to parental playfulness in the interaction with their children. In further studies, it could be useful to measure the effective parental playfulness and its effect on the trajectories of children’s playfulness, as parental and adults’ playfulness correlate with each other but are not congruent [43].

Lastly, future studies could explore the effects of parental playfulness and parental playtime on the individual dimensions of playfulness.

### 4.6. Implications

The study provides important lessons for practice in both home environments and pedagogical settings for children with low EFs. It highlights the role of parent’s playfulness and the time they spend playing with their children, particularly for those with low EF. This underlines how important parents are as role models for their children in their everyday lives. Because play is a natural part of what children do, parents who foster their children’s playfulness harmonize with their children’s inherent predilections. This is especially important for children with low EFs because it means that interventions should be integrated into children’s everyday lives to be optimally effective. 

### 4.7. Conclusions

Children with low EFs and children with EFs within the normal range differ significantly in how playful they are. This divergence persists longitudinally. Furthermore, children with low EFs seem to benefit particularly from playful parents, as these children also have higher levels of playfulness. Moreover, parental playtime with their children improves playfulness over time in children with low EFs. Parental play interactions seem to be particularly beneficial for children with low EFs if the parents act as role models and the support of children’s playfulness takes place in their daily home play environment. 

## Figures and Tables

**Table 1:** Demographic characteristics.

	Children with Low EFs	Children with EFs within the Normal Range
Mean Age in Years *(SD)*	5.39 (1.13)	5.45 (1.05)
Gender (Females/Males)	17/45	29/33
*Educational Setting*		
Childcare Center	17	17
Kindergarten	45	45
EF Score at T1 *(SD)*	−1.98 (0.85)	−0.04 (0.48)
*Family Language*		
German	38	44
Other	14	8
Unknown	10	10
*Nationality*		
Swiss	37	41
Other	15	11
Unknown	10	10
*Maternal Education*		
No Graduation	13	3
Professional	22	20
Academic	10	26
Unknown	17	13

Note. *SD* = standard deviation.

**Table 2:** Means and standard deviations for the children with low EFs and the children with EFs within the normal range at each time point on all the outcome variables.

	Children with Low EFs			Children with EFs within the NormalRange		
	T1 *M (SD)*	T2 *M (SD)*	T3 *M (SD)*	T1 *M (SD)*	T2 *M (SD)*	T3 *M (SD)*
Total playfulness score	3.65 (0.62)	3.69 (0.68)	3.67 (0.57)	3.92 (0.48)	3.98 (0.44)	3.94 (0.47)
Cognitive spontaneity	3.59 (0.81)	3.58 (0.91)	3.60 (0.70)	3.94 (0.62)	3.94 (0.65)	3.88 (0.61)
Social spontaneity	3.21 (0.85)	3.31 (0.87)	3.40 (0.80)	3.61 (0.58)	3.69 (0.50)	3.73 (0.60)
Physical spontaneity	4.02 (0.71)	4.00 (0.74)	3.85 (0.68)	4.03 (0.66)	4.20 (0.63)	4.06 (0.67)
Sense of humor	3.30 (0.85)	3.37 (0.93)	3.37 (0.78)	3.70 (0.63)	3.80 (0.53)	3.79 (0.55)
Manifest joy	4.13 (0.57)	4.18 (0.61)	4.12 (0.50)	4.30 (0.49)	4.40 (0.45)	4.27 (0.51)
Parental playfulness	4.51 (1.42)	N.A.	N.A.	N.A.	N.A.	N.A.
Parental playtime with their child	3.57 (1.02)	N.A.	N.A.	N.A.	N.A.	N.A.

*Note.* N.A. = not applicable; *M* = mean; *SD* = standard deviation.

**Table 3:** Intercorrelations between all the study variables for the children with EFs within the normal range (upper diagonal) and the children with low EFs (lower diagonal).

		1	2	3	4	5	6	7
1	Total playfulness score T1	-	0.68 ***	0.37 **	N.A.	N.A.	0.33 **	−0.01
2	Total playfulness score T2	0.74 ***	-	0.51 ***	N.A.	N.A.	−0.04	0.08
3	Total playfulness score T3	0.70 ***	0.63 ***	-	N.A.	N.A.	0.10	0.12
4	Parental playfulness T1	0.18	0.17	0.30	-	N.A.	N.A.	N.A.
5	Parental playtime with their child T1	−0.31 *	−0.45 **	−0.18	0.25	-	N.A.	N.A.
6	Age	−0.15	−0.14	−0.06	0.10	−0.18	-	−0.00
7	Gender ^1^	0.09	0.08	0.10	−0.22	−0.12	−0.07	-

*Note.* ^1^ female = 1; male = 0; N.A. = not applicable; * *p* < 0.05; ** *p* < 0.01; *** *p* < 0.001.

**Table 4:** Repeated measures-hierarchical linear model predicting children’s total playfulness score and its five dimensions at T3.

	Total Playfulness Score T3	Physical Spontaneity T3	Cognitive Spontaneity T3	Social Spontaneity T3	Sense of Humor T3	Manifest Joy T3
	*β* (SE)	*β* (SE)	*β* (SE)	*β* (SE)	*β* (SE)	*β* (SE)
Intercept	3.88 *** (0.08)	4.12 *** (0.10)	3.85 *** (0.10)	3.49 *** (0.10)	3.70 *** (0.11)	4.24 *** (0.07)
Group ^1^	−0.25 ** (0.09)	−0.13 (0.11)	−0.29 ** (0.11)	−0.32 ** (0.12)	−0.40 *** (0.12)	−0.12 (0.08)
T2	0.07 (0.04)	0.10 (0.06)	−0.01 (0.06)	0.10 (0.06)	0.12 * (0.06)	0.02 (0.05)
T3	0.04 (0.05)	−0.02 (0.06)	−0.05 (0.07)	0.16 ** (0.06)	0.11 (0.06)	−0.01 (0.05)
Age	−0.02 (0.04)	−0.05 (0.05)	−0.02 (0.05)	−0.02 (0.05)	−0.01 (0.06)	−0.01 (0.04)
Gender ^2^	0.06 (0.09)	−0.10 (0.11)	0.15 (0.12)	0.19 (0.12)	−0.00 (0.12)	0.07 (0.08)
R^2^ (fixed effects)	0.06	0.02	0.06	0.08	0.07	0.02
R^2^ (total)	0.66	0.59	0.58	0.66	0.67	0.44

*Note.* ^1^ children with low EFs = 1, children with EFs within the normal range = 0; ^2^ female = 1; male = 0; * *p* < 0.05; ** *p* < 0.01; *** *p* < 0.001.

**Table 5:** Repeated measures-hierarchical linear model including a time × group interaction predicting children’s total playfulness score and its five dimensions at T3.

	Total Playfulness Score T3	Physical Spontaneity T3	Cognitive Spontaneity T3	Social Spontaneity T3	Sense of Humor T3	Manifest Joy T3
	*β* (SE)	*β* (SE)	*β* (SE)	*β* (SE)	*β* (SE)	*β* (SE)
Intercept	3.87 *** (0.08)	4.06 *** (0.10)	3.86 *** (0.11)	3.51 *** (0.11)	3.69 *** (0.11)	4.26 *** (0.08)
Group ^1^	−0.24 * (0.10)	−0.01 (0.13)	−0.31 * (0.13)	−0.36 ** (0.13)	−0.38 ** (0.14)	−0.15 (0.10)
T2	0.07 (0.06)	0.18 * (0.08)	−0.02 (0.09)	0.07 (0.08)	0.12 (0.08)	−0.02 (0.08)
T3	0.05 (0.06)	0.09 (0.08)	−0.07 (0.09)	0.12 (0.08)	0.14 (0.08)	−0.02 (0.08)
T2 × Group ^1^	−0.01 (0.09)	−0.17 (0.12)	0.02 (0.13)	0.05 (0.12)	−0.01 (0.12)	0.07 (0.11)
T3 × Group ^1^	−0.03 (0.09)	−0.22 (0.12)	0.04 (0.13)	0.08 (0.12)	−0.06 (0.12)	0.01 (0.11)
Age	−0.02 (0.04)	−0.05 (0.05)	−0.02 (0.05)	−0.02 (0.05)	−0.01 (0.06)	−0.01 (0.04)
Gender ^2^	0.06 (0.09)	−0.10 (0.11)	0.15 (0.12)	0.19 (0.12)	−0.00 (0.12)	0.07 (0.08)
R^2^ (fixed effects)	0.06	0.03	0.06	0.08	0.08	0.02
R^2^ (total)	0.66	0.60	0.58	0.66	0.67	0.43

*Note.* ^1^ children with low EFs = 1, children with EFs within the normal range = 0; ^2^ female = 1; male = 0; * *p* < 0.05; ** *p* < 0.01; *** *p* < 0.001.

**Table 6:** Repeated measures-hierarchical linear model predicting children’s total playfulness score at T3 for children with low EFs depending on parental playfulness and parental playtime with their child.

	Total Playfulness Score T3
	*β* (SE)
Intercept	3.65 ***
T2	0.08
T3	0.10
Parental playfulness T1	0.13 *
Parental playtime with their child T1	−0.23 ***
Age	−0.09
Gender ^1^	−0.00
R^2^ (fixed effects)	0.18
R^2^ (total)	0.74

Note. ^1^ Female = 1; Male = 0; * *p* < 0.05; *** *p* < 0.001.

**Table 7:** Repeated measures-hierarchical linear model predicting children’s total playfulness score at T3 in children with low EFs depending on the interaction between time x parental playfulness as well as time × parental playtime with their child.

	Total Playfulness Score T3
	*β* (SE)
Intercept	3.69 ***
T3	0.06
Parental playfulness T1	0.13 *
Parental playtime with their child T1	−0.28 ***
T3 × parental playfulness T1	−0.00
T3 × parental playtime with their child T1	0.16 **
Age	−0.09
Gender ^1^	0.00
R^2^ (fixed effects)	0.19
R^2^ (total)	0.76

Note. ^1^ female = 1; male = 0; * *p* < 0.05; ** *p* < 0.01; *** *p* < 0.001.

## Data Availability

Dataset available upon request from the corresponding author.
